# Spontaneous Fracture in Phosphorus Workers

**Published:** 1899-08-19

**Authors:** 


### SPONTANEOUS FRACTURE IN PHOSPHORUS WORKERS.

Dr. W. F. Dearden[1] draws attention to the occurrence of fragilitas ossium in workers among phosphorus. He says that although, in considering the dangers arising from the use of yellow phosphorus in the manufacture of lucifer matches, prominence has always been given to the liability to necrosis of the jaw, this is not the only important pathological condition incidental to this industry, there being grounds for believing that a certain condition is brought about in the bony tissue as a result of which fractures of the long bones are very easily produced. He gives cases illustrating the condition, and refers to the recently issued report to the Home Secretary on the Use of Phosphorus in the Manufacture of Lucifer Matches, in which a special note is made of the experience of Dr. Boocoorens in Belgium, who "met with and treated in twenty-three years 30 cases of spontaneous fracture of the long bones caused by muscular effort, affecting exclusively the lower limbs of workmen who had been employed for years in dangerous departments and who had also suffered from necrosis of the jaw." The same sort of thing has also been reported by Dr. Kocher, of Berne, and Dr. Garman, of Bow Road.

[1] Brit. Med. Jour., July 29.

